# Supplementary material for: New Insights into Xanthophylls and Lipidomic Profile Changes Induced by Glucose Supplementation in the Marine Diatom Nitzschia laevis
Source: Mar Drugs. 2022 Jul 14;20(7):456. doi: 10.3390/md20070456 (PMC9318829; doi:10.3390/md20070456)
Supplement: Supplementary file 1 [file marinedrugs-20-00456-s001.zip › Supplementary file 2.pdf]

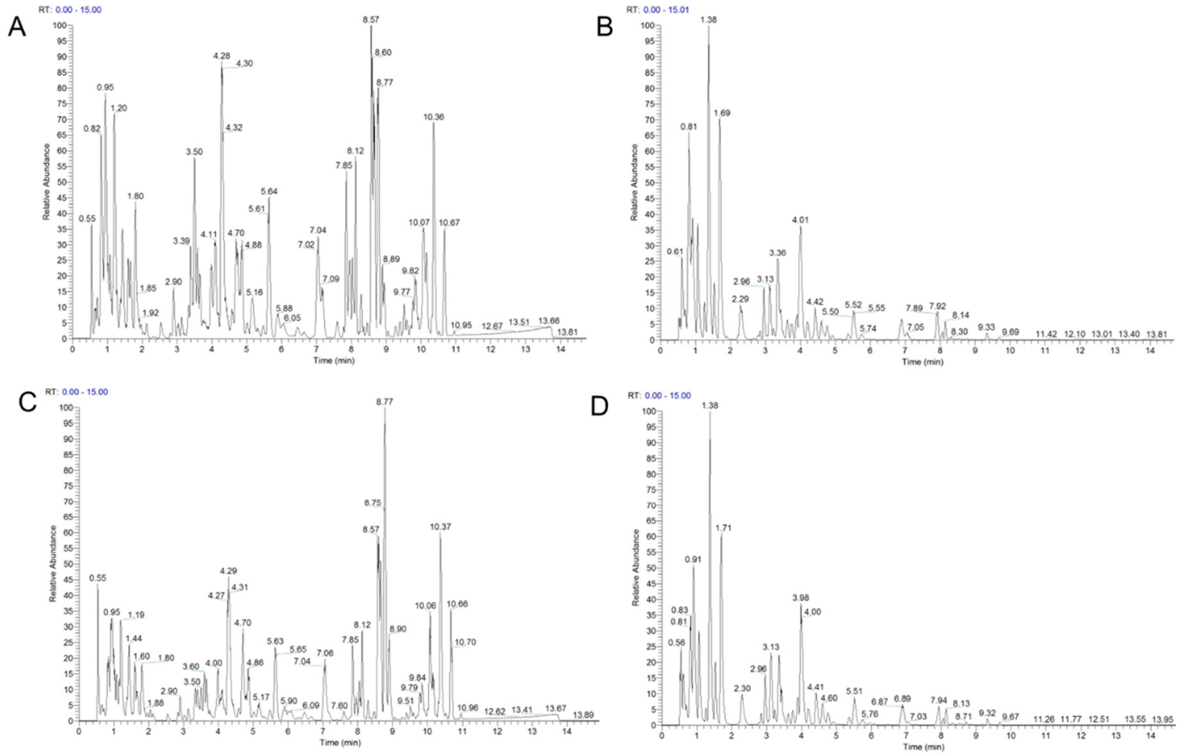

Figure S1. The chromatograms of base peak ion for the autotrophic culture (AC) ((**A**) for positive mode and (**B**) for negative mode) and mixotrophic culture (MC) ((**C**) for positive mode and (**D**) for negative mode).
